# Bone Marrow-Derived Progenitor Cells Augment Venous Remodeling in a Mouse Dorsal Skinfold Chamber Model

**DOI:** 10.1371/journal.pone.0032815

**Published:** 2012-02-28

**Authors:** Megan E. Doyle, Jeffrey P. Perley, Thomas C. Skalak

**Affiliations:** Department of Biomedical Engineering, University of Virginia, Charlottesville, Virginia, United States of America; University of California Merced, United States of America

## Abstract

The delivery of bone marrow-derived cells (BMDCs) has been widely used to stimulate angiogenesis and arteriogenesis. We identified a progenitor-enriched subpopulation of BMDCs that is able to augment venular remodeling, a generally unexplored area in microvascular research. Two populations of BMDCs, whole bone marrow (WBM) and Lin^−^/Sca-1^+^ progenitor cells, were encapsulated in sodium alginate and delivered to a mouse dorsal skinfold chamber model. Upon observation that encapsulated Sca-1^+^ progenitor cells enhance venular remodeling, the cells and tissue were analyzed on structural and molecular levels. Venule walls were thickened and contained more nuclei after Sca-1^+^ progenitor cell delivery. In addition, progenitors expressed mRNA transcript levels of chemokine (C-X-C motif) ligand 2 (CXCL2) and interferon gamma (IFNγ) that are over 5-fold higher compared to WBM. Tissues that received progenitors expressed significantly higher protein levels of vascular endothelial growth factor (VEGF), monocyte chemotactic protein-1 (MCP-1), and platelet derived growth factor-BB (PDGF-BB) compared to tissues that received an alginate control construct. Nine days following cell delivery, tissue from progenitor recipients contained 39% more CD45^+^ leukocytes, suggesting that these cells may enhance venular remodeling through the modulation of the local immune environment. Results show that different BMDC populations elicit different microvascular responses. In this model, Sca-1^+^ progenitor cell-derived CXCL2 and IFNγ may mediate venule enlargement via modulation of the local inflammatory environment.

## Introduction

Microvascular adaptations occur through the processes of angiogenesis, arteriogenesis, and venule remodeling in response to both physiological and pathological stimuli. Understanding how the microvasculature responds to external stimuli is critical if these processes are to be manipulated in the treatment of disease. Delivery of cells, specifically bone marrow-derived cells (BMDCs), is actively investigated as a means to stimulate the growth of new vasculature or enlargement of pre-existing vessels [1], [2]. The known effects of BMDCs on angiogenesis and arteriogenesis in the microcirculation supports the use of these cells for therapeutic benefit [1], [3], [4], [5]. The impact of these cells on the venular side of these microvascular networks, however, is less established.

The local delivery of BMDCs has become a widely investigated tool for the treatment of ischemic disease including peripheral limb ischemia and myocardial infarction [6], [7], [8], [9]. Additionally, delivery of BMDCs is also being investigated with regard to acceleration of wound healing, to specifically translate to clinical applications where diseases like diabetes may impede the body's ability to repair itself [10], [11], [12]. Whole bone marrow (WBM), bone marrow mononuclear cells (BM-MNCs), and purified groups of bone marrow-derived progenitor cells such as hematopoietic stem cells (HSCs), endothelial progenitor cells (EPCs) and mesenchymal stem cells (MSCs) have all been used to stimulate the restoration of blood flow in ischemic tissues via stimulation of new capillary growth or collateral vessel enlargement [13], [14], [15], [16]. Exogenous BMDCs are most often delivered to tissue via local cell injection, but some studies have used implantable matrices to deliver these cells in an effort to improve cell retention in the tissue [17], [18].

Once delivered, BMDCs have been shown to augment remodeling in tissues through a variety of mechanisms including transdifferentiation, cell fusion, support via paracrine mechanisms, and modulation of endogenous progenitor populations in the tissue [2], [19], [20], [21], [22], [23]. Recent work also shows that cytokines released from BMDCs can modulate inflammatory function by enhancing the recruitment of circulating leukocytes to sites of exogenous cell delivery [24], [25]. Upon arrival in the tissue, these inflammatory cells are also able to modulate remodeling via growth factor production.

In this study, the *in vivo* mouse dorsal window chamber model was used in conjunction with a variety of experimental techniques including intravital image analysis, immunohistochemistry, gene expression profiling, and protein level quantification to evaluate microvascular remodeling responses on both structural and molecular levels. The dorsal skinfold window chamber enables chronic observation of the microcirculatory process of wound healing. Specifically, this model allows for repeat analysis of remodeling vasculature and allows for the application of cells directly to the surface of the remodeling vasculature.

Two populations of BMDCs were compared with regard to their ability to affect microvascular remodeling. Whole bone marrow (WBM) and a Lin^−^/Sca-1^+^ subfraction of bone marrow (Sca-1^+^ progenitor cells) were chosen to specifically represent two main components of the adult bone marrow: the mature inflammatory cells and the undifferentiated progenitor cells. The Lin^−^/Sca-1^+^ subpopulation of cells includes a majority of the aforementioned progenitor subsets including HSCs, EPCs, and MSCs.

Cell encapsulation in sodium alginate was used as a delivery method because it allows for cells to be placed in close proximity to the remodeling microvessels and can help maintain the presence of delivered cells in the tissue since the internal structure of the gel inhibits mobility of the cell population, limiting cell migration out of the porous matrix [26], [27].

Upon initial observation that Sca-1^+^ progenitor cells encapsulated in sodium alginate enhance venular remodeling, we sought to investigate this phenomenon further by evaluating how and why venules enlarge in response to these cells. Analysis of gene expression, protein levels, and immunohistochemistry have enabled us to hypothesize a putative mechanism by which these delivered cells are able to augment venular remodeling in the dorsal skinfold chamber model.

## Methods

### Ethics Statement

The following animal procedures were performed in compliance with the US Department of Health and Human Services Guide for the Care and use of Laboratory Animals and were approved by the Animal Care and Use Committee of the University of Virginia under protocol number 3467.

### Dorsal Skinfold Window Chamber

C57Bl/6 male mice between 24 and 28 grams were anesthetized with an intraperitoneal injection of ketamine (0.03 mL), xylazine (0.015 mL), and atropine (0.005 mL) diluted in 0.2 mL of saline. Under sterile conditions, a circular section 12 mm in diameter was surgically excised exposing the microcirculation within the hypodermis. A window chamber was then surgically implanted around the dissected circle of tissue and covered with a round coverglass. Mice were allowed 24 hours to recover from surgery before BMDC implantation.

### Bone Marrow-Derived Cell Isolation and Encapsulation

Bone marrow was isolated from the femur, tibia, and hip bone in each hindlimb of 25–30 g male C57Bl/6 mice. Bones were ground in a mortar and pestle in Phosphate Buffered Saline (PBS) with 2% fetal bovine serum (FBS) and filtered through a 100 µm cell strainer (BD). The WBM solution was centrifuged and resuspended in sodium alginate solution for encapsulation, or PBS with 2% FBS and 5% Normal Rat Serum for progenitor cell separation. The Lin^−^/Sca-1^+^ fraction of the WBM was isolated using magnetic isolation using both hematopoietic progenitor cell enrichment and biotin selection kits (Stem Cell Technologies). WBM or Lin^−^/Sca-1^+^ cells were mixed in a 1.5% w/v alginate solution and aspirated into 102 mmol/L CaCl_2_ solution (in saline). Newly formed alginate beads (1 mm) contained 2×10^4^ cells. Prior to bead implantation, cell viability was checked with a live/dead assay (Invitrogen). Once viability over 80% was verified, one 1 mm bead was carefully placed in the center of each dorsal skinfold chamber 24 hours following surgery (Day 1). **Table 1** outlines the experimental groups used for this portion of the study.

**Table 1:** Bone Marrow-Derived Cell Delivery and Control Groups.

Treatment	n
WBM	15
Sca-1+Progenitors	15
Vehicle	11
Untreated	11

### Encapsulated Cell Delivery and Intravital Image Acquisition

At each time point, mice were anesthetized using 2.5% isoflurane. Prior to imaging, the glass coverslip was removed from the window and adenosine (10^−4^ M) in Ringer's solution was topically applied to the tissue for ten minutes to maximally dilate vessels in the chamber. The entire window was imaged at 4× magnification on days 1, 4, 7 and 10, and montages were constructed using Adobe CS2 image processing software. Image J software (NIH) was used to measure all vascular metrics. Vessels are distinguished as arterioles or venules based on blood flow direction at the time of imaging. In reconstructed images, diameters were measured at a distance two vessel diameters away from vessel branch points. A measure of blood column width was made for every vessel and vessel branch that could be distinguished at all timepoints. Starting vessel diameters for arterioles and venules were divided into two bins, above and below the mean (30 um and 57 um, respectively), so that the responses of comparably sized vessels were compared to one another.

### Tissue Harvest and Immunohistological Staining

Following image acquisition on day 10, window chamber tissues were perfusion fixed, harvested, and embedded for frozen sectioning as previously described [28]. Embedded tissues were sectioned at a thickness of 15 µm. Some slides were stained with Hematoxylin and Eosin (H&E) while others were labeled with immunofluorescent antibodies. Slides were labeled with 1∶200 smooth muscle α-actin 1A4 (Sigma), 1∶50 biotin conjugated CD45 (BD Pharmingen), or 1∶20 MCP-1 (eBioscience). Slides were then mounted with VectaStain hard mount containing DAPI for labeling of cell nuclei (Vector Labs).

Fluorescently labeled specimens were examined using confocal microscopy. All measurements were made in a blinded fashion. Each section stained for CD45 was imaged with a consistent gain-setting at 20× in three areas of the section. The number of CD45 positive cells and total cell nuclei were manually counted for each area. Additionally, the number of CD45 positive cells and number of total nuclei were manually counted at 20× magnification in large venules within the SM α-actin positive medial walls, and 10 µm beyond the medial layer to account for inflammatory cells present in the adventitia. In these images, the luminal circumference of SM α-actin expression was used to normalize the number of CD45^+^ cells per vessel. Whole H&E labeled sections were imaged at 50× magnification. Venules were analyzed for wall area and number of nuclei per venule. These metrics were normalized by vessel luminal or inner circumference since imperfect cross-sections prevent normalization by vessel diameter.

### Gene Expression Profiling in Whole Bone Marrow and Sca-1^+^ Progenitors

Real time reverse transcriptase PCR (RT-RTPCR) was conducted using a PCR array to analyze the expression of a focused panel of genes related to the promotion and inhibition of angiogenesis. The array was used to profile the gene expression of 84 growth factors and inhibitors in isolated WBM and Sca-1^+^ progenitor cells. WBM was treated as a control cell type for comparison with the progenitors. A conservative threshold of five-fold was set to identify genes that are expressed at substantially different levels among the two cell types. RNA was extracted from WBM and Lin^−^/Sca-1^+^ cells following cell separation using TRIzol (Invitrogen) and retrotranscribed using a RT^2^ First Strand Kit (SABiosciences). PCR was performed using the Angiogenic Growth Factors and Angiogenesis Inhibitors RT^2^ Profiler PCR Array and RT^2^ SYBR Green/Fluorescein qPCR Master Mix (SABiosciences). Gene expression levels for WBM and Lin^−^/Sca-1^+^ cells were calculated with the ΔΔCt method with normalization to the average expression level of five housekeeping genes. Then, relative differences in gene expression between WBM and Sca-1^+^ progenitors were evaluated. A positive value indicates elevated gene expression in progenitor cells relative to WBM. PCR arrays were run twice for each cell population using RNA isolates from two different cell separation procedures. Each Sca-1^+^ progenitor RNA sample was prepared with cells harvested from eight animals, and each WBM RNA sample was prepared with cells harvested from four animals.

### Quantification of Cytokine Expression in Mouse Window Chamber Tissues

Following image acquisition on day 10, animals were euthanized with pentobarbital sodium (60 mg/kg i.p.). At days 1, 4 and 10 the window area was dissected away from the dorsal area and homogenized in RIPA buffer (Sigma) containing protease and phosphatase inhibitors (Roche). Prior to performing ELISAs (R&D Systems), total protein levels in all samples were determined using a protein assay with a bovine serum albumin standard (Bio-Rad).

### Statistical Analysis

One-Way Analysis of Variance (ANOVA) was used in experiments where multiple groups were present. If ANOVA analysis determines a difference between groups, the Student-Newman-Keuls method was used as a post hoc test to determine significance below p = 0.05. For experiments where only two groups were present, comparisons were made using Student t-tests. All statistical analysis was performed using SigmaStat software and values are presented +/− SE.

## Results

### Sca-1^+^ Progenitors Cells Augment Venular Remodeling

**Figure 1:**
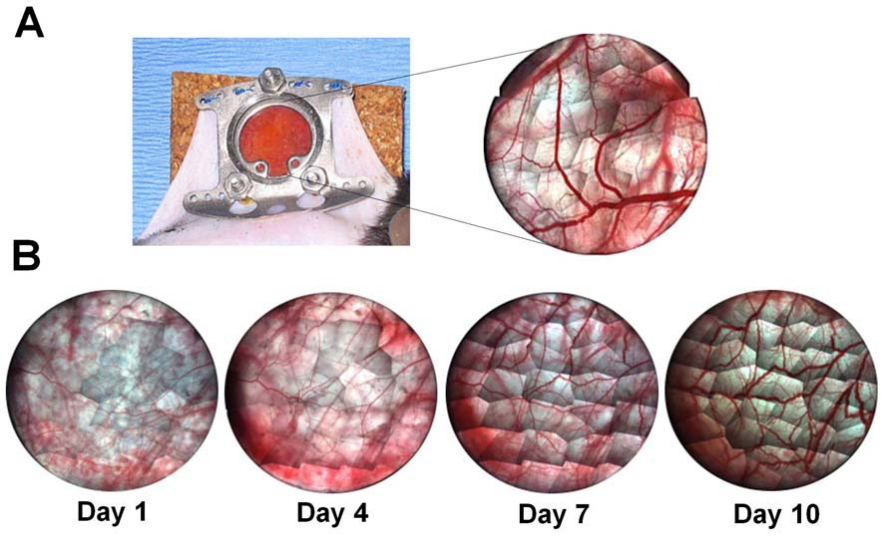
Microvascular Remodeling in the Mouse Dorsal Window Chamber. (A) Representative montage of the window chamber at 4x. (B) Example ten day time course of the remodeling network. Arterioles and venules enlarge slightly from days one to four. Diameter increases and angiogenic remodeling is accelerated from days 4 to 10.

The mouse dorsal skinfold window chamber model was used to evaluate the impact of delivered BMDCs on microvascular remodeling (**Figure 1**). Vessels within the skinfold chamber underwent angiogenesis, arteriogenesis, and venular remodeling over time, occurring as a result of the inflammatory stimulus of the surgery alone (**Figure 1B**).

**Figure 2:**
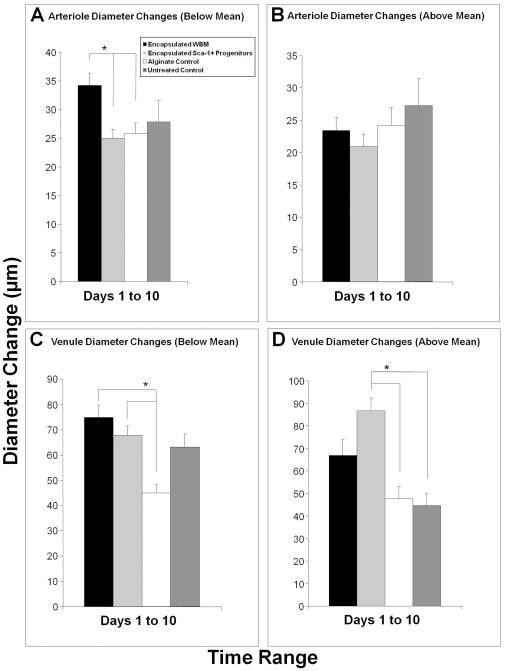
Encapsulated Cells' Impact on Arteriole and Venule Diameters over 10 Days. (A) Average absolute arteriole diameter change over ten days in vessels with starting diameter below the mean. (B) Average absolute arteriole diameter change over ten days in vessels with starting diameter above the mean. (C) Average absolute venule diameter change over ten days in vessels with starting diameter below the mean. (D) Average absolute venule diameter change over ten days in vessels with starting diameter above the mean. * indicates p<0.05 as calculated by ANOVA. Data are means +/− S.E.

In arterioles with starting diameters below the mean, encapsulated WBM elicited an arteriogenic response in the chamber that exceeded the response in both encapsulated Sca-1^+^ progenitor recipients and alginate control construct recipients (**Figure 2A**). This result supports the hypothesis that WBM can have an arteriogenic impact on remodeling vasculature. However, in this model, the arteriogenic response observed in the smaller arterioles does not exceed that of the untreated control (**Figure 2A**).

BMDCs had a significant impact on venular remodeling (**Figure 2C**), specifically Sca-1^+^ progenitor cells on the larger venules in the tissue (**Figure 2D**). In venules with starting diameters above the mean, encapsulated Sca-1^+^ progenitor cell recipients underwent diameter changes from Day 1 to Day 10 that surpassed both alginate and untreated controls by 81% and 93%, respectively (**Figure 2D**).

These results suggest a previously undiscovered role for Sca-1^+^ progenitor cells in venular remodeling. We sought to investigate this observation with further analysis into the structural and molecular adaptations taking place in the tissue in response to the Sca-1^+^ progenitor cell delivery.

### Venular Structural Adaptations in Response to Delivered Sca-1^+^ Progenitor Cells

**Figure 3:**
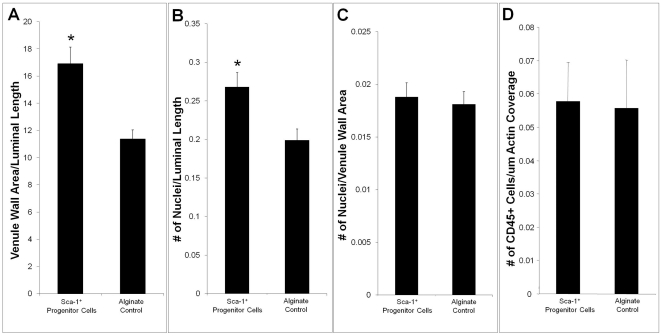
Structural Analysis and Cellular Composition of Remodeled Venules. (A) Average venule wall area per micron inner luminal circumference. (B) Number of nuclei in venule wall per micron inner luminal circumference. (C) Number of cell nuclei per square micron venule wall area. (D) Number of CD45 positive cells per micron luminal actin length. * indicates p<0.05 as calculated by Student's t-test. Data are means +/− S.E.

H&E histological analysis was used to assess venous structural adaptations in animals that received encapsulated Sca-1^+^ progenitor cells or alginate control constructs (**Figure 3**).

Final venule wall area was 49% larger in the animals that received encapsulated Sca-1^+^ progenitor cells (**Figure 3A**). Additionally, there were more cell nuclei in the walls surrounding venules in progenitor recipients (**Figure 3B**). However, the number of cell nuclei per square micron of venule wall area was comparable among both groups (**Figure 3C**). Since the number of cells per area in the vessel walls was comparable between venules from animals that received encapsulated progenitor cells and animals that received only an alginate control construct, immunohistochemical analysis was used to evaluate whether the thickened walls were a result of increased inflammatory cell investment. Immunohistochemical analysis revealed, however, that the number of CD45^+^ inflammatory cells in the venule walls was also comparable in both groups (**Figure 3D**).

### Sca-1^+^ Progenitor Cells Express High Levels of Angiogenic Activator mRNA

Encapsulated Sca-1^+^ progenitor cells were able to elicit a strong venular remodeling response in the dorsal skinfold chamber. To achieve better mechanistic understanding of the role that these cells play in the remodeling process, potential molecular mediators were evaluated with a PCR array designed to analyze the expression of a focused panel of genes related to the promotion and inhibition of angiogenesis.

**Figure 4:**
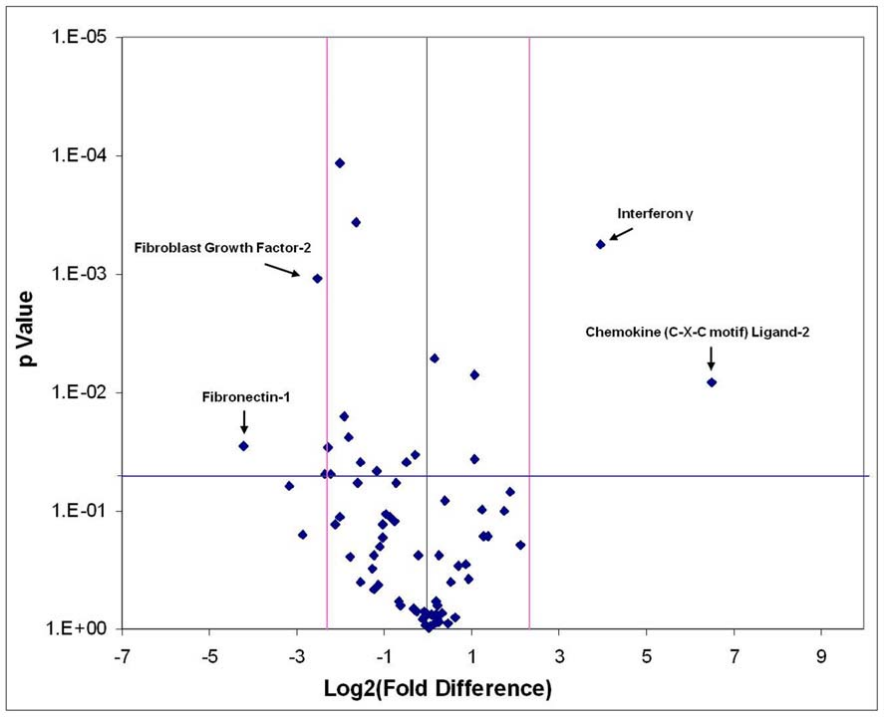
Statistical Analysis of mRNA Transcripts from Multiple Cell Isolations. Fold differences between mRNA transcript levels were compared for two isolations of the WBM and progenitor populations. The black line indicates no fold-change in gene expression. Pink lines indicate a five-fold change in gene expression threshold. The blue line indicates a threshold of statistical significance indicated by p<0.05 as calculated by Student's t-test.

Of the 84 genes compared in multiple isolations of both cell types, two were reproducibly expressed over five-fold higher in WBM and two were reproducibly expressed over five-fold higher in the Sca-1^+^ progenitors (**Figure 4**).

Of these, mRNA transcripts of fibroblast growth factor-2 (FGF-2) and fibronectin-1 (Fn-1) were expressed at much higher levels in WBM cells compared to Sca-1^+^ progenitors (5.05 fold and 18.39 fold higher, respectively). Two factors, interferon gamma (IFNγ) and chemokine (C-X-C motif) ligand-2 (CXCL2), contained transcripts at much higher levels in the Sca-1^+^ progenitors (15.44 fold and 91.39 fold higher, respectively).

**Figure 5:**
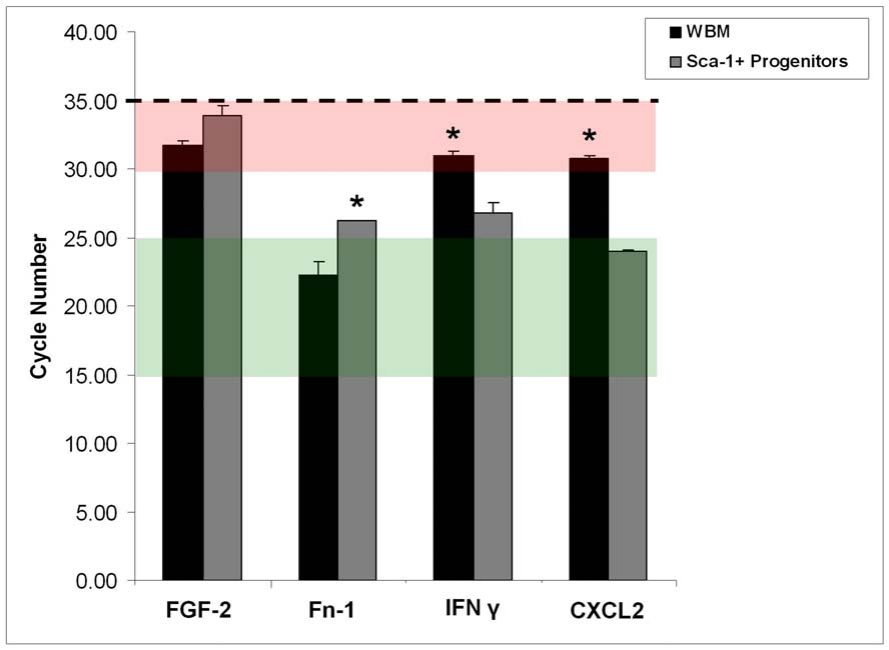
Comparison of Negative Threshold to Cycle Number of Gene Amplification for WBM and Sca-1^+^ Progenitors. Fn-1, IFN γ, and CXCL2 amplify at cycles significantly below the negative threshold in both WBM and Sca-1^+^ progenitors, while FGF-2 amplifies at a cycle number indicative of low genetic expression in both cell types. * indicates p<0.05 as calculated by ANOVA. Data are means +/− S.E.

Next, gene expression was analyzed relative to a negative threshold cycle number. Genes that amplify at cycle numbers above 35 were considered non-amplifying, indicating very low to no expression (**Figure 5**). The red box indicates the range of cycle numbers where gene expression is considered very low, and the green box shows the range where genes would be considered early amplifying and very abundant. In both cell types, FGF-2 amplified at a cycle number that is indicative of very low genetic expression. In WBM, IFNγ and CXCL2 both amplified at cycle numbers that signify very low expression of these cytokines. However, in Sca-1^+^ progenitors both of these genes amplified at cycle numbers that are indicative of high genetic expression. Fibronectin-1 did not appear to be a low amplifying gene in either cell type, although its expression was significantly higher in WBM cells.

### Dorsal Tissues Express Elevated Vasculogenic Cytokines in Response to Sca-1^+^ Progenitor Cell Delivery

ELISAs were used to determine the tissue levels of vascular endothelial growth factor (VEGF), platelet-derived growth factor-BB (PDGF-BB), monocyte chemotactic protein-1 (MCP-1), epidermal growth factor (EGF), tumor necrosis factor-α (TNF-α), and chemokine (C-X-C motif) ligand 2 (CXCL2/MIP-2) within whole window chamber tissues. These proteins were chosen because previous work suggests that they could play a role in venular remodeling [29], [30], [31], [32], [33], [34], [35], [36], [37]. The expression of each of these factors can also be elicited by progenitor cell secretion of CXCL2 or IFNγ [38], [39].

**Figure 6:**
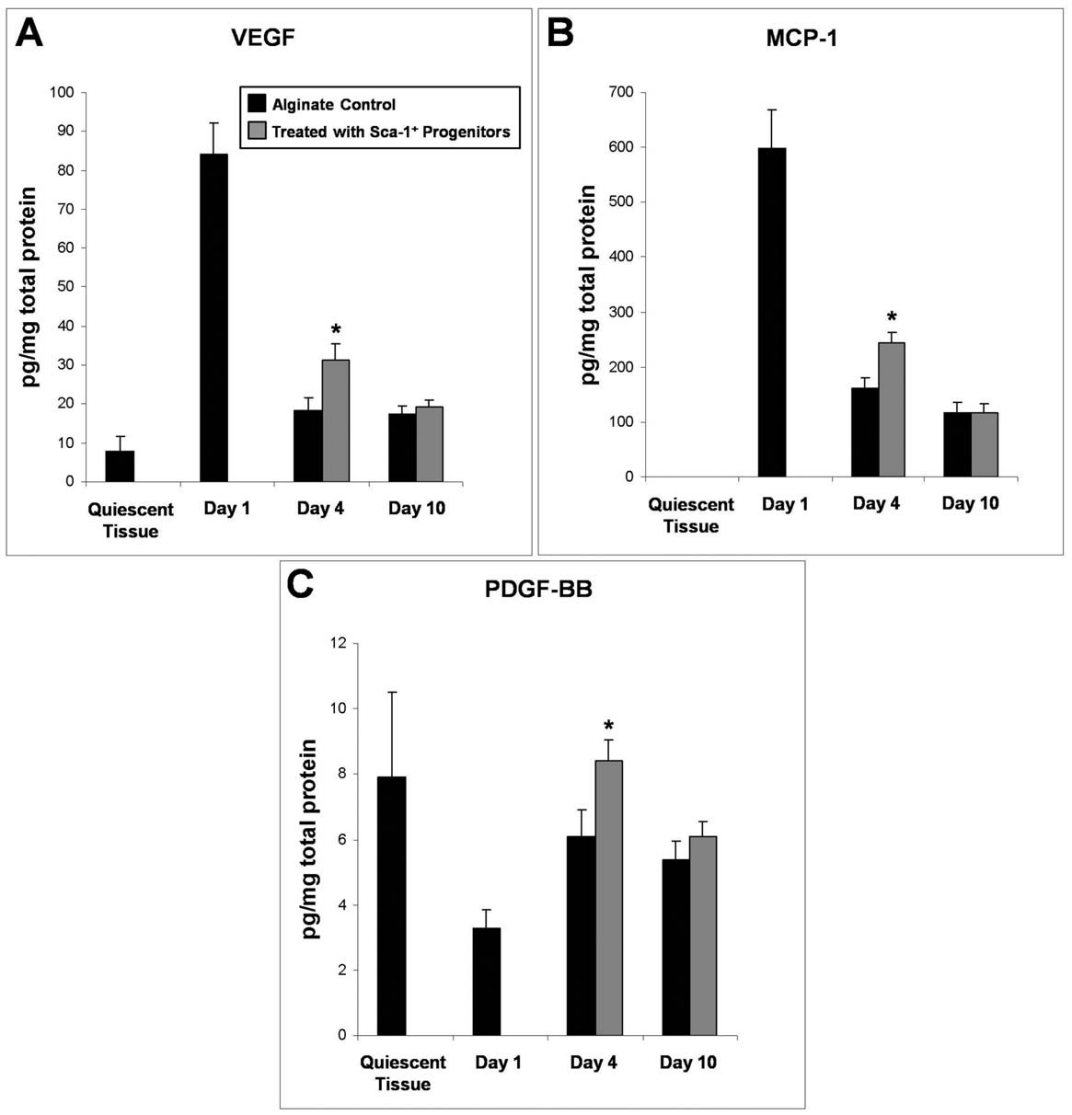
Enhanced Protein Expression of VEGF, MCP-1, and PDGF-BB in Dorsal Skinfold Chambers. (A) Delivery of encapsulated Sca-1^+^ progenitor cells elevated VEGF protein expression in dorsal tissue at day 4. (B) Delivery of encapsulated Sca-1^+^ progenitor cells elevated MCP-1 protein expression in dorsal tissue at day 4. (C) Delivery of encapsulated Sca-1^+^ progenitor cells elevated PDGF-BB protein expression in dorsal tissue at day 4. * indicates p<0.05 as calculated by ANOVA. Data are means +/− S.E.

Elevated expression of VEGF and MCP-1 over that of quiescent tissue was observed at Day 1 and is likely a byproduct of the initial inflammatory response to the surgical implantation of the dorsal skinfold chamber. In addition, protein levels of VEGF, MCP-1, and PDGF-BB were all significantly elevated in progenitor cell treated animals at Day 4 (**Figure 6**). VEGF protein expression in Sca-1^+^ progenitor recipients was 1.7 fold higher (**Figure 6A**), and MCP-1 and PDGF-BB protein expression in Sca-1^+^ progenitor recipients was 1.4 fold higher relative to alginate control animals (**Figure 6B,C**). Expression of EGF, TNF-α, and CXCL2/MIP-2 was comparable between Sca-1^+^ progenitor and alginate control-treated tissues (data not shown).

### Delivery of Encapsulated Sca-1^+^ Cells Elevates the Number of Tissue Resident Inflammatory Cells

**Figure 7:**
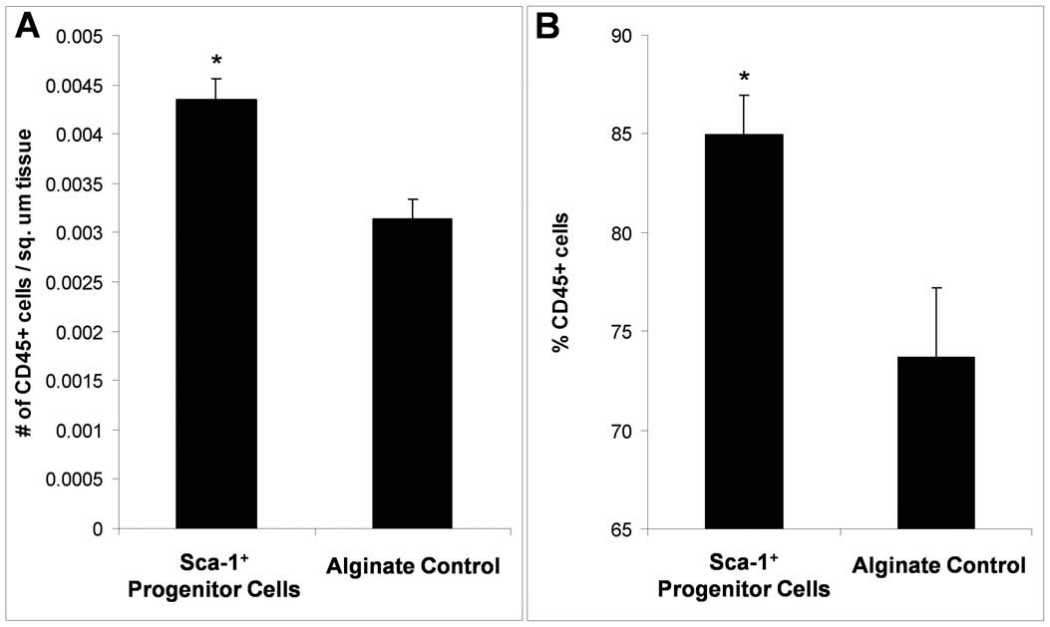
Inflammatory Cell Recruitment in Sca-1^+^ Progenitor Cell Recipients and Control Animals. (A) Number of CD45 positive cells per square micron skinfold chamber tissue. (B) Total percentage of CD45 expressing cells in the skinfold chamber tissue. * indicates p<0.05 as calculated by ANOVA. Data are means +/− S.E.

Next, to evaluate whether inflammatory cell recruitment could be playing a role in the observed venular remodeling in response to Sca-1^+^ progenitor cells, tissue sections from animals that received either encapsulated Sca-1^+^ progenitors or alginate-only constructs were analyzed for differences in CD45^+^ cell density (**Figure 7A,B**).

At day 10, the density of CD45^+^ cells in the window chamber tissue of Sca-1^+^ progenitor cell recipients was elevated compared to alginate control recipients (**Figure 7A**). These new inflammatory cells appear to make up a significant contribution to the dorsal tissue since the percentage of CD45^+^ cells to total cells in the tissue is also significantly elevated in the Sca-1^+^ cell recipients (**Figure 7B**).

**Figure 8:**
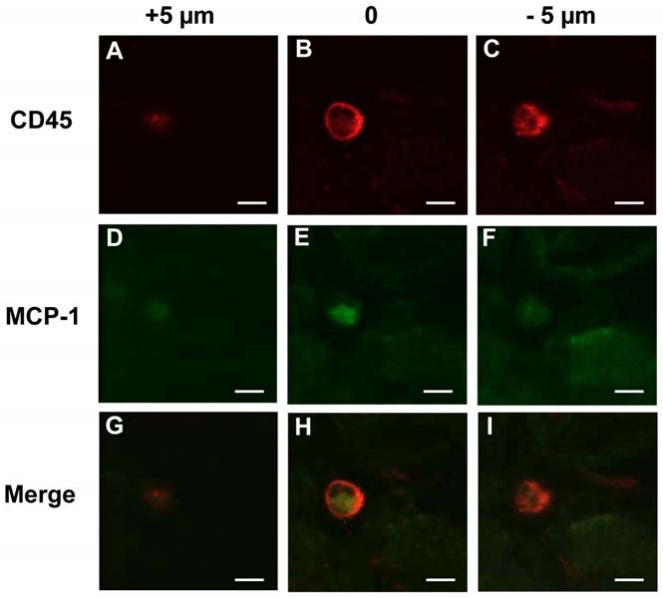
MCP-1 Expression in CD45^+^ Cells in Sca-1^+^ Progenitor Cell Recipients. (A–C) CD45 expression in a cell 5 microns above and below the focal plane. (D–F) MCP-1 expression in a cell 5 microns above and below the focal plane. (G–H) Merged CD45 and MCP-1 expression in a cell 5 microns above and below the focal plane. (Scale bar, 5 µm).

The elevated number of CD45^+^ cells in the animals that received Sca-1^+^ progenitors suggests that these cells could be primary mediators of the observed venular enlargement and that they accomplish this through the production of multiple growth factors including VEGF, MCP-1, and PDGF-BB. In order for the CD45^+^ cells to accomplish this, however, they must be able to produce these growth factors within the window chamber tissue. Therefore, immunohistochemical analysis was used to confirm that the CD45^+^ cells are able to produce MCP-1 in the chamber. To analyze MCP-1 antibody expression in these cells, serial Z-sections were taken at 5 µm intervals to confirm protein expression to be localized within CD45^+^ cells (**Figure 8**). The detected presence of MCP-1 within the CD45^+^ cells supports the hypothesis that they are capable of contributing to the elevated levels of VEGF, MCP-1, and PDGF-BB measured within the chamber at Day 4 (**Figure 6**).

## Discussion

Results of this study suggest that delivered BMDCs can influence venular remodeling and identify possible molecular mechanisms by which progenitor-enriched BMDCs are able to elicit venular structural adaptations. Previous work has identified venular remodeling in response to electrical stimulation, vein grafting, hypertension, and allogenic endothelial cell matrix implantation [40], [41], [42], [43], [44]. To date, however, venular remodeling in response to BMDC delivery is either not quantified or is grouped together with vessel enlargements on the arteriole side of the network. Observation of venular enlargement in response to delivered Sca-1^+^ progenitor cells is not entirely surprising since similar populations of cells have been shown to promote enhanced collateralization in an ischemic hindlimb model [6], [19]. Endothelium and smooth muscle on venules, like their arteriolar counterparts, have the ability to modulate their response to a variety of stimuli [40], [41], [42], [43], [44].

In response to biomechanical and biochemical stimuli, venules can alter their structure by increasing their luminal diameter, and exhibit increases in inflammatory and smooth muscle cell wall investment [41], [45]. We observed that in response to Sca-1^+^ progenitor cell delivery the walls of these enlarged venules were thickened and contained increased numbers of cell nuclei. However, the number of cells per area in the vessel walls was comparable between venules from animals that received encapsulated progenitor cells and animals that received only an alginate control construct, suggesting that the thickened walls are a result of increased cell investment. While the number of CD45^+^ inflammatory cells was elevated in the tissue surrounding the vessels, the number of these cells in or closely associated with the venule walls was comparable in both groups. Thus, the cells responsible for increasing venule wall thickness in Sca-1^+^ progenitor cell recipients may be mural cells like smooth muscle or pericytes. This is not surprising since in progenitor cell-treated tissues we quantified an elevated level of PDGF-BB, a cytokine that upregulates the proliferation of smooth muscle and pericytes [46]. Previous work has shown that in the mouse dorsal skinfold window chamber model, venule structural diameter is increased in response to PDGF-BB growth factor delivery [31]. Thus, PDGF-BB may be a primary mediator of venular structural remodeling in this model.

**Figure 9:**
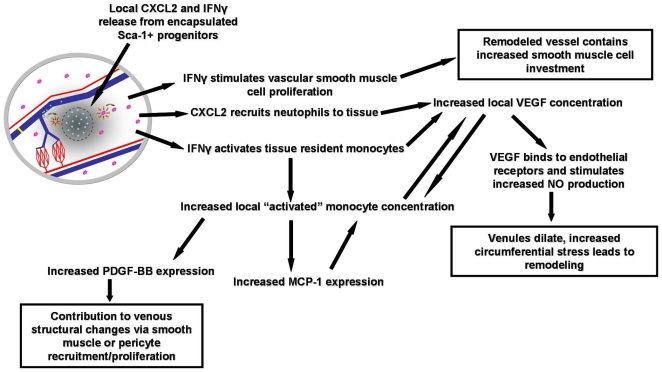
Proposed Mechanism of Venular Remodeling in Response to Sca-1^+^ Progenitor Cells. Processes by which CXCL2 and IFNγ derived from delivered Sca-1^+^ progenitor cells may stimulate the remodeling of venules in the dorsal skinfold window chamber. Boxes highlight processes that have led to venular structural adaptation.

We have shown that the Sca-1^+^ subfraction of bone marrow is sufficient in stimulating venule remodeling in the skinfold chamber, and we have observed that WBM does not elicit the same effect in the tissue. To begin to understand why these two cell populations differ with regard to enhancing venular remodeling, we sought to identify differences in the expression of angiogenic cytokines among the two populations. In keeping with other studies of BMDCs, gene expression analysis of these populations revealed elevated expression of many growth factors and angiogenic cytokines that are known to modulate inflammatory and angiogenic processes [2], [47], [48], [49], [50]. However, analysis of these populations of cells revealed that both CXCL2 and IFNγ were very highly expressed in the Sca-1^+^ progenitors compared to WBM. Thus, we believe that in this model these two cytokines may be responsible for modulating the dynamic process that ultimately results in enhanced venular remodeling. By integrating this information with data from other published studies, we can begin to construct a possible mechanism by which these cells elevate tissue expression of VEGF, PDGF-BB, and MCP-1 and enhance venular remodeling (**Figure 9**).

Past research has shown that IFNγ can act directly on vascular smooth muscle to stimulate proliferation and remodeling [51]. This factor can also activate tissue resident monocytes that are able to increase local expression of VEGF and PDGF-BB as well as recruit additional activated monocytes to the dorsal tissue, via MCP-1 expression [39]. Studies have demonstrated that the stimulation of neutrophils with CXCL2 leads to their recruitment and release of biologically active VEGF-A that initiates an angiogenic cascade within the affected tissue [38]. Elevated concentration of VEGF can stimulate vasodilation by acting on endothelial receptors to increase nitric oxide production by endothelial cells [52]. Nitric oxide could then relax smooth muscle on the venules, increasing circumferential wall stress. This stimulus has been linked to arteriole remodeling and might impact venules similarly [53], [54]. Finally, elevated levels of PDGF-BB can enhance pericyte and smooth muscle cell migration and proliferation that may lead to increased mural cell investment in the venular walls [46].

Our observation of elevated transcript levels of CXCL2 and IFNγ in Sca-1^+^ progenitor cells and our understanding of the mechanisms by which these factors can lead to the remodeling of vessels led us to hypothesize that delivered Sca-1^+^ progenitors are able to modulate the local inflammatory response within the dorsal tissue. It is possible that the delivered Sca-1^+^ progenitor cell production of CXCL2 and IFNγ is not large enough to elevate whole tissue levels of either factor since the number of delivered cells is very small when compared to the total number of cells within the dorsal skinfold chamber. However, growth factor secreted from delivered cells may be substantial enough to impact native cells in the skinfold chamber local to the implanted cell/alginate construct. Once these native cells become activated, they can elevate their production of growth factors and adhesion molecules, including VEGF, MCP-1 and PDGF. The increased production of these factors can also stimulate the recruitment of additional inflammatory cells to the tissue. These inflammatory cells can contribute to cytokine production in the dorsal tissue and additional cell recruitment. This positive feedback process would lead to a significant elevation of inflammatory cells and growth factor production in the tissues that received progenitor cells.

The possibility of inflammatory cell modulation by the delivered Sca-1^+^ progenitors was evaluated by quantifying the number of CD45^+^ cells in the tissue at day 10. Compared to control tissues, Sca-1^+^ progenitor cells increased inflammatory cell presence in the tissue by 39%. We also confirmed that these CD45^+^ cells are a potent source of MCP-1 by showing cellular expression of this factor in tissue from Sca-1^+^ progenitor recipients. In addition to MCP-1, these cells are also capable of producing high levels of VEGF and PDGF-BB [55], [56]. The idea that delivered cells may affect microvascular remodeling by modulating the local inflammatory environment is a concept that is garnering more attention. Recent work has shown that transplanted MAPC-derived progenitor cells increase tissue levels of SDF-1, CXCL2, and MCP-1 and that these cells elevate the recruitment of CD11b^+^ cells to the ischemic hindlimb [24].

Overall, this study provides new information regarding molecular regulation in the still under-explored area of venular remodeling and provides insight into the complex interactions between transplanted, local inflammatory, and vascular cells within a remodeling tissue. Understanding these behaviors is critical for successful implementation of cell therapy in the clinical setting.
